# Biomimetic Aspects of Oral and Dentofacial Regeneration

**DOI:** 10.3390/biomimetics5040051

**Published:** 2020-10-12

**Authors:** Akshaya Upadhyay, Sangeeth Pillai, Parisa Khayambashi, Hisham Sabri, Kyungjun T. Lee, Maryam Tarar, Stephanie Zhou, Ingrid Harb, Simon D. Tran

**Affiliations:** McGill Craniofacial Tissue Engineering and Stem Cells Laboratory, Faculty of Dentistry, McGill University, 3640 University Street, Montreal, QC H3A 0C7, Canada; akshaya.upadhyay@mail.mcgill.ca (A.U.); sangeeth.pillai@mail.mcgill.ca (S.P.); parisa.khayambashi@mail.mcgill.ca (P.K.); hisham.sabri@mail.mcgill.ca (H.S.); kungjun.lee@mail.mcgill.ca (K.T.L.); maryam.tarar@mail.mcgill.ca (M.T.); stephanie.zhou@mail.mcgill.ca (S.Z.); ingrid.harb@mail.mcgill.ca (I.H.)

**Keywords:** biomimetics, dentistry, dentofacial, regeneration

## Abstract

Biomimetic materials for hard and soft tissues have advanced in the fields of tissue engineering and regenerative medicine in dentistry. To examine these recent advances, we searched Medline (OVID) with the key terms “biomimetics”, “biomaterials”, and “biomimicry” combined with MeSH terms for “dentistry” and limited the date of publication between 2010–2020. Over 500 articles were obtained under clinical trials, randomized clinical trials, metanalysis, and systematic reviews developed in the past 10 years in three major areas of dentistry: restorative, orofacial surgery, and periodontics. Clinical studies and systematic reviews along with hand-searched preclinical studies as potential therapies have been included. They support the proof-of-concept that novel treatments are in the pipeline towards ground-breaking clinical therapies for orofacial bone regeneration, tooth regeneration, repair of the oral mucosa, periodontal tissue engineering, and dental implants. Biomimicry enhances the clinical outcomes and calls for an interdisciplinary approach integrating medicine, bioengineering, biotechnology, and computational sciences to advance the current research to clinics. We conclude that dentistry has come a long way apropos of regenerative medicine; still, there are vast avenues to endeavour, seeking inspiration from other facets in biomedical research.

## 1. Introduction

The term “biomimetics” was derived from the Greek words “*bios*” (meaning life) and “*mimesis*” (meaning to imitate). It originally meant developing any new material or technology that mimics nature or is obtained from nature. In biology, biomimetics relates to harnessing bioinspired materials or molecules, either synthetic replacements of natural structures or derivations from living organisms that simulate biological mechanisms. The field of tissue engineering and regenerative medicine (TERM) has developed significantly over the past decade with the main focus on the synthesis of novel, highly intricate biomaterials and techniques to regenerate and replace lost structures. However, in light of the human body’s complex anatomy and functions, it has always remained a challenge to develop state-of-the-art, accurate, bio-replacements for different tissues and organs. The craniofacial region is residence to living paradoxes, with hardest to the softest tissues, and involves a macromolecular to nanomolecular range of therapies. Each tissue and organ has their own peculiarities that have to be dealt with to achieve the utmost biological resemblance to natural tissues. Biomimetic dentistry has come a long way in engineering and regenerating dental hard and soft tissues unprecedentedly. We hypothesize that biomimetic improvements are highly essential in successfully engineering dentofacial structures. This current review provides a glimpse of the volumes of work that has been done over the past decade to improve these aspects. The purpose of this study is to establish a guide to new researchers, clinicians, and dentists at all stages of research to help them develop a perspective of biomimetics and its importance in clinical therapies, specifically in restorative dentistry, oral and maxillofacial surgery, and periodontology.

The literature was searched on Medline (OVID) with key terms like “biomimetics”, “biomaterials”, and “biomimicry” combined with MeSH terms for “dentistry”, and a limit was set to 2010–current, where over 500 articles were obtained under clinical trials, randomized clinical trials, metanalysis, and systematic reviews together. Out of the obtained search, relevant clinical studies have been included with systematic reviews, along with hand-searched preclinical studies as potential therapies wherever deemed necessary. A brief discussion on enamel regeneration and recent outlooks in pulp regeneration has been covered. Pulp tissue is a complex connective tissue with multiple functions including protective, nutritive, and reparative activities. Infection to pulp due to caries or trauma usually results in complete pulp removal. This makes the tooth structure more fragile and prone to fracture. Therefore, it is important to use the most biomimetically advanced material in these situations to preserve or replace the pulp tissue to save the tooth structure. We have discussed the most significant biomimetic analogues for tooth structure and different scaffolds for dentin pulp complex regeneration. Further, we have focused on the current preclinical and clinical studies in orofacial bone regeneration and a brief overview of strategies for repair of the oral mucosa under oral and maxillofacial dentistry. As important as the tooth structure, it is also essential to understand the supporting periodontal structure, which are the hard and soft tissue that surround the tooth that undergoes competitive regeneration processes. An array of different tissue engineering strategies and surgical methods are used today to induce regeneration of the complex highly cellular periodontium, and thus, in the last section of this review, we cover the current facets in periodontal tissue engineering, including cell and cell-free approaches and guided tissue regeneration, with a brief description of implant biomimetics.

## 2. Biomimetics in Restorative Dentistry

### 2.1. Enamel Biomimetics

**Figure 1:**
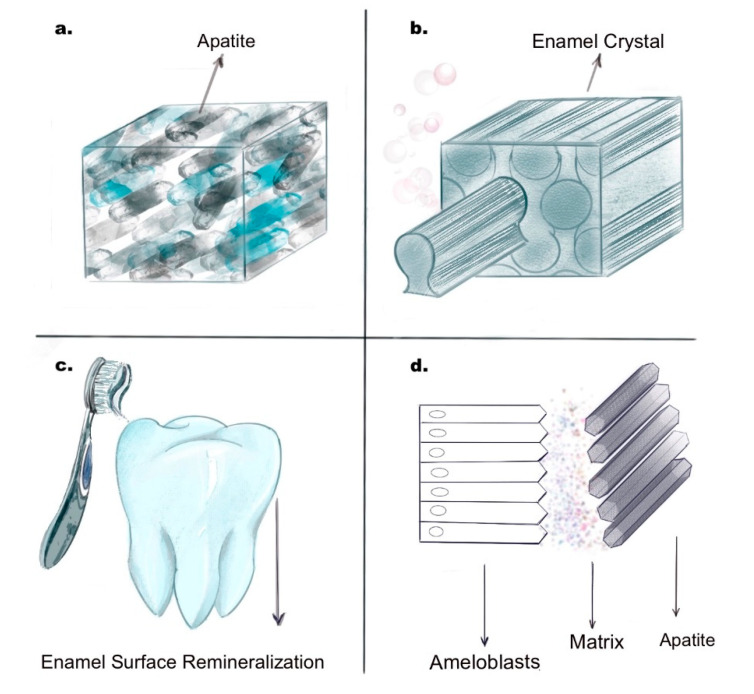
Mechanisms of enamel tissue engineering and regeneration: (**a**) physiochemical synthesis of apatite crystals, (**b**) protein-matrix-guided enamel crystal development, (**c**) enamel surface mineralisation using fluoride toothpastes, and (**d**) ameloblast (cell-based) tissue engineering of synthetic enamel apatite. Image adapted from [3] Pandya, M.; Diekwisch, T.G.H. Enamel biomimetics-fiction or future of dentistry. *Int. J. Oral Sci.* **2019**, *11*, 8. Copyright 2019 Springer Nature.

Enamel is the outermost hard tissue covering the crown of the tooth structure. It is considered the hardest substance in the body due to its high inorganic content (96%), mostly comprised of interwoven hydroxyapatite crystals arranged in a three-dimensional pattern, giving it superior aesthetic and structural properties [1]. However, continuous and complex changes occurring within the oral microenvironment sometimes lead to enamel demineralisation, thereby initiating caries formation. Dental caries affects more than two-thirds of the world’s population and is highly prevalent among people of all ages. The origination of caries is contributed by a multitude of factors including the presence of cariogenic bacteria, dietary carbohydrates, decreased salivary flow, or xerostomia. Usually, there is a balance between the demineralisation and remineralisation processes in the oral cavity, but this equilibrium is lost due to factors consistently favouring tooth demineralisation, leading to primary white spot lesions, caries progression, and eventually cavitation [2]. Proper teeth cleaning to get rid of cariogenic bacteria, adequate salivary flow, and the presence of sufficient amounts of calcium and phosphate ions in saliva can help control the limit of tooth demineralisation to a certain extent. However, since the body’s natural defence might not be enough to resist caries, in many cases, minimally invasive dentistry approaches are used in a desperate attempt to remove initial caries and to preserve as much of the natural tooth structure to maintain the functional integrity and aesthetics of the tooth. Nevertheless, enamel regeneration still remains a challenging task, and it becomes even more complex on clinical implementation. Therefore, it is essential to look at alternate methods for enamel repair and engineer biomaterials that mimics the natural enamel both biologically and structurally. Pandya et al. (2019) described four different pathways for enamel tissue engineering and regeneration (Figure 1) by (a) physiochemical synthesis, (b) protein-matrix-guided enamel crystal development, (c) enamel surface remineralisation, and (d) cell-based regeneration [3]. We will discuss these approaches with their most recent advances in enamel mimetics.

#### 2.1.1. Physiochemical Synthesis

Biomimetic substitutes developed to replace the natural tooth enamel are synthesized using extreme conditions to simulate the natural enamel structure, which includes use of high temperature and pressure [4]. Chen et al. (2005) first described the possible development of synthetic hydroxyapatite nanocrystals, which mimicked the enamel prism-like structures [4]. Their study showed how a combination of aqueous hydroxyapatite and docusate sodium salt, when adjusted to a pH of 5.5, led to precipitation of long apatite crystals around 200–400 nm in size. Further, to limit these rods’ size, the hydroxyapatite aqueous solution was replaced with a fluorapatite solution and processed under extreme hydrothermal pressures, leading to enamel rods with a size around 5–10 micron matching the natural enamel size (Figure 1a). Fan et al. (2009) developed controlled remineralisation of enamel hydroxyapatite crystals using amelogenin and fluoride with a newly developed methodology to sequentially form fluoridated needle-like enamel crystals. The biomimetic solution was prepared using commercially obtained calcium and phosphates at 2.5 mM and 1.5 mM, respectively, at 37 °C with 50 mM trihydroxymethylaminomethane-hydrochloric acid and 180 mM NaCl buffer at 7.6 pH. Fluoride concentration from NaF was kept at 1 mg/L to form the desired solution, and previously obtained tooth slices were treated with 3% HNO_3_ solution for 50 seconds followed by immersion in the biomimetic solution. Scanning electron microscopy (SEM) results showed the formation of flake-like fused crystals on the enamel’s surface, which was porous in structure. CaP (calcium phosphate) nanorods formed were around 25 nm in diameter and 100 nm in length [5]. A few years later, an approach established by Ren et al. (2012) used a sodium bicarbonate buffer solution and a mixture containing calcium nitrate tetrahydrate, sodium bicarbonate, disodium hydrogen phosphate, and octa calcium phosphate, all maintained at a pH of 6.6, resulting in the formation of human enamel-like crystals with a size ranging between 100–500 nm [5]. An essential modification applied to their crystallisation approach was the use of high temperatures (150–200 °C) constantly for 72 h. However, the pH and pressure range were maintained at standard conditions. More recently, Wang et al. (2017) described a three-step process to form enamel to mimic the natural enamel formation sequalae. First, they conjugated the carboxymethyl chitosan (CMC) with a bisphosphonate alendronate, which stabilised the amorphous calcium phosphate (ACP) to form a CMC/ACP nanoparticle complex. The second step used a sodium hypochlorite solution to break the CMC/ACP nanoparticles formed in the first step. Once the nanoparticles were degraded, glycine was added to orient the ACP/CMC to form a well-organized and spatially arranged enamel rods. This technique is notable as it simulates the biologic amelogenesis process and supports the initial layering of amelogenin matrix protein, leading to cell–matrix and matrix–crystal interactions and subsequently to the elongation of apatite crystals [6].

#### 2.1.2. Protein-Matrix-Guided Synthesis

Enamel engineering using protein matrix formation aims to biologically mimic the natural process of tooth enamel development. It involves the synthesis of amelogenin-rich proteins followed by their conjunction with calcium and phosphate ions. This technique usually involves enzymatic processing with enamel proteases like matrix metalloproteases 20 (MMP 20) and kallikrein 4, which provide a three-dimensional axis for crystal growth [3] (Figure 1b). The three major proteases that pave ways for enamel matrix development are amelogenin, ameloblastin, and enamelin [7]. During amelogenesis, each matrix protein plays a vital role in enamel matrix layering and the consequent crystal growth. Some earlier studies showed the use of octacalcium phosphate solutions along with amelogenin (10% *w*/*v*) to initiate crystal formation [8]. Similar studies using other combinations of enamel matrix proteins with octacalcium resulted in a more organized, better apatite crystal structure formation [9]. However, these combinations did not completely mimic the natural enamel structure in terms of their arrangement, hardness, or strength. More recently, studies focussed on understanding how the amelogenin amino acid chains are formed and the mechanism of their interaction with developing enamel crystals [10]. For example, Li et al. (2014) described the interaction between a leucine-rich 59 peptide amelogenin synthesized using an alternate splicing technique, which showed a tendency to form spheroidal nanoparticles arranged in a linear pattern [11]. With the development and understanding of better biomimetic materials like chitosan, a chitosan gel-based combination of amelogenin fragments with MMP 20 added to calcium phosphate crystal solution was studied and tested. This led to the breakdown of amelogenin fragments after primary crystal formation with improved biomechanical properties for the subsequently formed apatite crystals [12]. However, even today, most of these methods only manage to focus on matrix formation and crystal formation as separate entities, and a more sophisticated, novel technique needs to be developed to mimic the in vivo aspects of enamel formation.

#### 2.1.3. Enamel Surface Mineralisation

As described earlier, enamel mineralisation is a complex and multifactorial process. However, excessive demineralisation leads to white spot lesions, which marks the initiation of caries. Several approaches have been tried over the years to induce surface remineralisation. The impacts of fluoridation on tooth structure has been investigated for over a century now, and the results clearly indicate their current applications in dentistry (Figure 1c). Fluoride ions usually move the bioavailable calcium and phosphates from saliva and lead to fluorapatite formation, which has superior resistance to enamel demineralisation and thereby limits caries progression [13]. Based on this principle, several innovative oral care products have been developed. Most of them include the use of fluoridated toothpastes and other fluoride-containing products like varnishes and mouthwashes. Casein phosphopeptide (CPP)–amorphous calcium phosphate (ACP) combinations are used for improving enamel surface remineralisation owing to their ability to stabilise calcium, phosphate, and fluoride ions, which are already available in saliva, leading to faster and better surface remineralisation of the enamel layer [14]. Ma et al. (2019) conducted a systematic review with metanalyses describing the efficiency of CCP–ACP in enamel remineralisation. They summarised 12 studies based on the inclusion and exclusion criteria, which were evaluated either by surface roughness or their microhardness values. The evaluation showed significant heterogenicity in the surface hardness values, due to which a random model of analysis was performed which included 5 of the 8 studies selected and which showed values (SMD = 1.19, 95% CI: [0.72, 1.66], *p* < 0.00001) indicating that the use of CPP–ACP resulted in superior remineralisation. The atomic force microscopy (AFM) analysis of three of their studies also showed that CPP–ACP’s use resulted in reduced roughness of the enamel surface and showed their ability to repair and form a smooth surface [14,15]. In another study by Fernando et al. which described the use of SnF2 along with ACP–CCP to induce tooth repair, their in-vitro studies showed the ability of SnF2 to interact with CPP–ACP complexes to form a nanofilament coating on the tooth surface, with superior remineralisation activity in comparison to either of these materials individually. The mechanism involves Sn2 to form cross-links with CPP–ACP to stabilise the bioavailable minerals and to thereby enhance binding of the ion binding to the tooth minerals. The results showed that this novel combination can help to significantly improve resistance to caries and dentinal hypersensitivity [15]. A study by Bossu et al. (2019) compared a biomimetic nanoparticle-infused hydroxyapatite toothpaste with two other toothpastes with different fluoride concentrations. Their focus was directed towards how nanoparticle-based HA integrated to enamel surface and formed a coating that is similar to natural enamel apatite structures. This technique avoids any physiochemical reaction between fluoride ions and enamel crystals and, at the same time, is more resistant to brushing abrasions and grindings due to superior chemical bond between the old enamel and new layer of apatite crystals formed [16]. These modifications provide better resistance to caries while prevent the risk of fluorosis due to the overuse of fluoride-based substituents. Use of bioactive glass for enamel white spot lesions have been studied extensively in the last few years [17]. These glass particles when in contact with physiological fluids has the ability to from new apatite crystals, thus essentially remineralising the enamel surface. Besides, these glasses when incorporated with fluoride formed the more resistant fluorapatite layering over enamel surface. This has allowed their use in toothpastes, varnishes, and dental cements to treat carious lesions. In a recent systematic review by Taha et al., they compared the efficiency of different toothpastes containing fluorides, ACP–CPP combinations and bioactive glass and evaluated the different studies showing efficiency of each material in improving white spot lesions [18]. Many studies showed the superior properties of bioactive glasses in forming a mineral layer on an enamel surface rich in calcium, phosphate, and silica [17]. Some studies showed the improved mechanical properties in newly formed enamel using bioactive glass-based toothpastes [19,20]. Based on these studies, bioactive glasses were ranked above both fluoride and casein peptidases in remineralising enamel white spot lesions and are an effective alternate option.

#### 2.1.4. Cell and Tissue Culture Systems for Enamel Organ Engineering

Enamel tissue engineering approaches ideally include complex interactions between enamel forming cells (chiefly ameloblasts) with biomimetic scaffolds and enamel proteins to form suitable in vitro conditions to engineer new enamel crystals (Figure 1d). Although cell- and tissue-based engineering approaches have been used for developing several organs and structures in the human body, enamel bioengineering still remains a daunting challenge due to the highly sensitive nature of the ameloblast cells and the inability to retrieve enamel organ stem cells with superior pluripotency as they are lost immediately after tooth eruption [21]. The lack of a suitable and more stable ameloblast cell line is another drawback in enamel tissue engineering. Currently available ameloblast cell lines usually rely on the feeder layer system to provide sufficient nutritional support or interaction between mesenchymal cells (feeder layer) to induce primary ameloblast cell growth. The currently available ameloblast cell line includes the mouse ameloblast-lineage cell line (ALC), the rat dental epithelial cell line (HAT-7), mouse LS8 cell line, porcine PABSo-E cell line [22], and the rat SF2-24 cell line [23]. The ALC is the oldest of all the cell lines and expresses amelogenin and tuftlins that are important markers, indicating their close relation to ameloblast-like cells. Even the other cell lines mentioned show ameloblastic characteristics, but each of them moreover focuses on one or other specific markers or areas of enamel formation and, therefore, still remains an insufficient tool to accurately simulate in vivo enamel development [3]. However, well-characterized, more specifically, human stem cell-derived cell lines in combination with supporting scaffolds and matrix proteins may help bridge the current knowledge gap and limitations in synthetic engineering enamel. On successfully developing such biomimetic enamel regenerative systems, the future of dental restorative therapies can be immensely benefited.

### 2.2. Biomimetic Aspects of Dentin and Dentin-Pulp-Complex Regeneration

Dentin forms the bulk of the tooth structure and is a highly complex tissue: 70% of the dentin structure is attributed to its mineral composition, and the remaining 30% is attributed to its organic content and water. In the tooth structure, there are of two types: (1) peripheral or mantle dentin and (2) circumpulpal dentin. The mantle dentin forms the hard outer layer of dentin, whereas the circumpulpal dentin, as its name suggests, makes up the larger part of the dentin and surrounds the entire pulp tissue [24]. The lower mineral content of the dentin biostructure makes them prone to quicker demineralisation than enamel. Its complex structure makes its remineralisation slower, and consequently, the spread of caries into dentin is enhanced, causing infection of the pulp tissue and, eventually, periapical diseases. In this context, it is essential to develop smart and effective biomaterials to replace lost tissue mineral and organic content to form dentinal tubules and to induce remineralisation. Several materials and bioactive analogues can induce this process; however, in this review, we discuss the key strategies for dentin remineralisation.

Use of resin-based adhesives and bioactive glass for dentin remineralisation is an established approach for caries affected or partially demineralised dentin, which involves the deposition of newly formed dentin on the previously carious dentin surfaces [25]. Bioactive glass (BAG) causes effective remineralisation of dentin and improves the mechanical properties of the dentin structure through intrafibrillar mineralisation. This allows to maintain the functionality of the tissue in addition to superior physical properties. However, in this type of dentin formation, lack of seed crystals or the old dentin crystals will affect the formation of new dentin. For these reasons, ion-based dentin regeneration may not be sufficient to remineralise dentin that has already been entirely demineralised by caries [26]. However, ion leaching or an ion releasing approach for dentin caries removal or remineralisation has been widely used (Table 1). It includes using several bioactive and biomimetic analogues which not only replace the lost mineral structure but also, at the same time, protect the collagen fibrils from degradation. The most commonly used biomimetic materials include bioactive glass (BAG), calcium silicates, calcium orthophosphates, and zinc oxide (ZnO) particles. Other non-ion-based biomaterials, which are developed more recently and overcomes the drawbacks of ion-based biomaterials, are described in Table 2.

### 2.3. Dentin-Pulp Complex Regeneration

**Figure 2:**
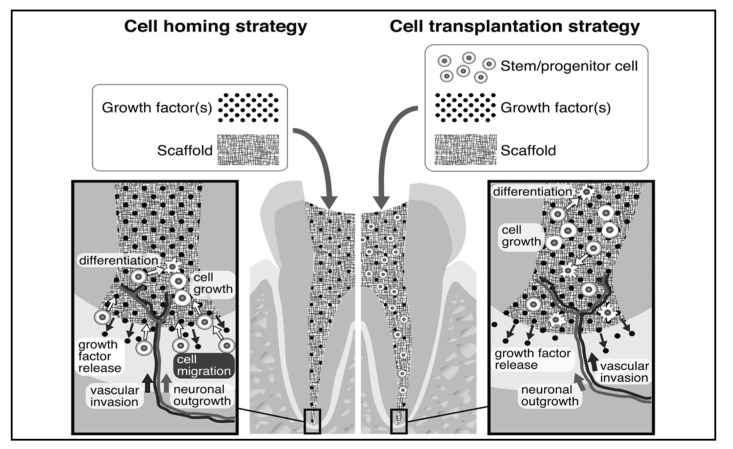
Strategies for dentin pulp complex regeneration: the cell homing strategy involves injection of growth factors and scaffolds into the pulp tissue, which leads to proliferation and migration of progenitor cells from the apical pulp tissue. In cell transplantation, the stem cells are injected to the pulp space along with growth factors and scaffolds to induce pulp regeneration. In both scenarios, cell growth or proliferation due to growth factors or peripheral induction is followed by scaffold colonization, which in cases with resorbable scaffolds leads to formation of new tissue over time. Image reprinted with permission from Morotomi et al. [64].

When carious lesions are left untreated, they extend beyond hard tissues and cause inflammation and, in turn, infection of the pulp and surrounding periapical tissues. In these situations, the treatment plan depends on pulp vitality. In vital pulp therapy, the principle is to induce dentinal bridge formation to protect the pulp tissue. In cases with a nonvital pulp tissue, till today, root canal treatment is the standard of care procedure which removes all necrosed or decayed pulp tissue to clean the tooth canals and to fill them with an inert obturating material which creates a three-dimensional seal both coronally and apically, thus preventing any microleakage and tooth structure preservation. However, easier said than done, endodontic treatments are associated with several posttreatment complications, including periapical pathologies, pain, and secondary infections due to incomplete debris removal or improper techniques used for pulp therapy [40,41]. A study by Prati et al. (2018) showed that, in a 20-year follow up study on post-root-canal-treated patients, 15% of subjects developed some form of periapical lesion and almost 21% of these cases ended in extraction of the tooth based on cumulative teeth survival statistics [42]. Therefore, it is evident from the previous studies that there are several drawbacks to the currently used pulp capping and root canal sealing materials, which dictates the need for more refined biomimetic materials with controlled signalling and a more directed differentiation of the pulp cells to limit inflammatory responses and to thus allow better healing. Several approaches are currently being used to fulfil the biologic and structural needs required to eventually preserve the pulp tissue and tooth structure. These include both cell-free (Table 3) and cell-based therapies (Table 4) for dentin pulp complex regeneration. Other methods employ a combination of cells and growth factors which are either directly transplanted to the pulp space (Figure 2) or are laden in combination with biomimetic scaffolds (Table 5) to induce pulp regeneration.

**Figure 3:**
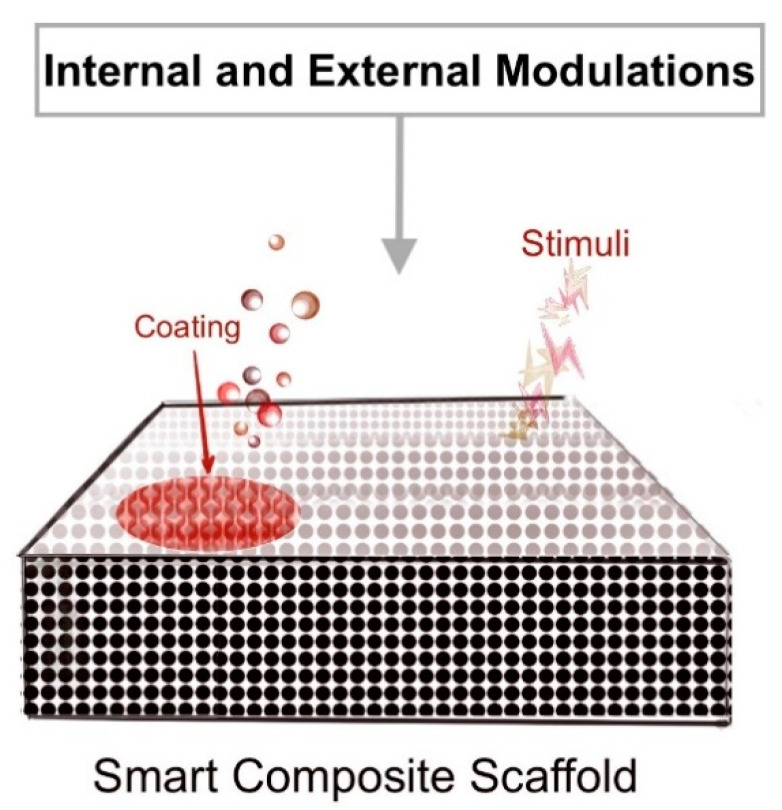
Smart scaffolds for dentin pulp regeneration. Image adapted from Perez et al. [118] and Moussa et al. [119].

Several materials have been developed and tested to evaluate their potential in dentin and pulp regeneration. So far, a large part of this research focused on comparing these biomaterials in both in vitro and in vivo studies to develop novel smart biomaterials (Figure 3) [118,119]. These materials are fabricated in a way that allows easy internal and external modifications based on the inflicted stimuli and thus fulfils all the biomimetic requirements necessary to initiate tooth hard and soft tissue regeneration. However, it has been observed that two biomaterials, Mineral trioxide aggregate (MTA) and CaOH, as well as their modifications were extensively studied in this context by independent researchers. Tabarsi et al. (2012) [120] studied the effect of MTA and Calcium enriched mixture (CEM) on rabbit dorsal skin. They used a fresh mixture of both the materials, which was randomly applied on the skin surface; washed off after 4 h; and evaluated the surface for erythema after 1, 24, 48, and 72 h. The results showed more erythema on the MTA surface as compared to CEM. On histological examination, MTA showed a higher inflammatory response as compared to CEM. Their study concluded that CEM is a more biocompatible material for endodontic procedures [120]. A randomized control trial compared CEM and MTA for pulpotomy treatments, which showed no significant differences in apexogenesis (root closure) radiographically after 12 months follow up [121]. However, Azimi et al. (2014) [122] performed a comparative study between MTA and bioceramic paste, where no statistically significant result was seen when evaluating the inflammatory response or hard tissue formation between both the materials after Cvek’s pulpotomy [122]. In one systematic review, the studies selected compared MTA with CaOH and tricalcium silicate and tested for their success rate, inflammatory response, and dentin bridge formation. They evaluated 46 studies and showed that MTA (odds ratio: 2.72) had a significantly higher success rate in all aspects when compared to CaOH. On the contrary, no noticeable difference was seen between MTA and tricalcium silicates (odds ratio 1.18) [123]. In another systematic review by da Rosa et al. (2018) [124], they compared over 716 papers and 83 patents, which mainly studied CaOH, followed by MTA. The study concluded that MTA surpassed CaOH in all aspects and was favourable for pulp regeneration [124]. MTA and Biodentine^TM^ were also compared by Celik et al. (2019) [125] as pulpotomy agents in pulp-exposed carious teeth. Their results show a 100% radiographic success rate after 24 months for MTA and 89.4% for biodentine^TM^ [125]. It is evident from these studies that, although the literature has a lot of evidence and data on the biomimetic properties of each biomaterial discussed here, it is still inconclusive when it comes to individual clinical implementation. Success in clinical dentin-pulp preservation or regeneration depends on the tooth, type of carious lesion, and pulp injury.

Although we have developed some highly bioactive and biocompatible materials as medicaments and scaffolds for dentin and pulp repair and regeneration, there is still a need to simultaneously organize both biological and mechanical aspects of these biomaterials. Our primary goal is to induce appropriate signalling pathways for cell–cell and cell–matrix interactions while being noncytotoxic to cells with superior mechanical properties that will mimic in vivo conditions to engineer the dentin pulp complex. Researchers should focus on developing tailor-made biomimetic analogues while keeping in mind the essentials of dentin pulp regeneration, including revascularization, cell, differentiation, and growth factor integration with the ability to induce good quality remineralisation of hard tissues.

## 3. Biomimetics in Oral and Maxillofacial Regeneration

### 3.1. Biomimetics in Bone Regeneration

#### 3.1.1. Bone, a Complex Hub, and a Multitasker

**Figure 4:**
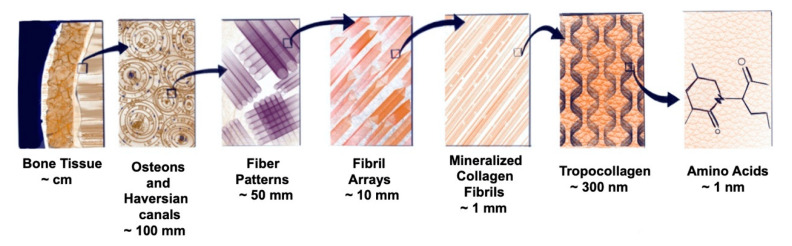
Macro- and microstructural arrangement of bone: the macroscale structure comprises of dense outer compact bone and spongy inner cancellous bone. Compact bone is arranged into osteons that form haversian canals. These osteons are formed by fibres arranged in geometrical patterns. These fibres are made up of collagen fibrils which have alternating organic phases to form fibril arrays. Each array makes up one collagen fibre. Collagen consists of protein molecules (tropocollagen) formed from three chains of amino acids. Image adapted from Launey et al. [135].

Bone is a highly specialised, complex, and dynamic part of the skeletal system. Apart from the major function of providing a framework for all the tissues, it is inherently involved in maintaining several physiological activities, namely haematopoiesis, regulation of ions, maintenance of muscle mass, and a lot more. It can be classified in two forms: trabecular (medullary) and compact bone (cortical), both having separate and distinct functions. Trabecular or spongy bone, as its name suggests, has larger pore size to accommodate hematopoietic cells and comprises the bone marrow, while cortical bone has more osteocytes and is involved in responding to mechanical signals by bone remodelling (mechanotransduction) [126,127,128]. Cortical bone is highly dense (less than 20% porosity) and composed of closely knit osteons which concentrically form cylindrical systems known as Haversian system, lodging a blood vessel in the centre (Figure 4). This system has anisotropic mechanical properties, with the modulus of elasticity (E) = 20 GPa along the Haversian system and E = 8 GPa along the transverse axis, thus providing a rigid structure [129,130]. On the other hand, cancellous bone has >90% porosity and is arranged into plates (trabeculae), which offers a larger surface area to mass ratio and better flexibility with E = 100 MPa [131]. Furthermore, bone comprises organic and inorganic components (collagen-hydroxyapatite matrix) that provide an interplay of elasticity and rigidity, respectively [132]. Increase in the collagen mineralisation increases the modulus of elasticity, which makes it possible to bear more stress. In contrast, pure collagen carries the capacity to bear deformation [133]. Furthermore, bone organization and regeneration by requisition of different molecules like collagen and growth factors are facilitated by extracellular components like glycosaminoglycans (GAGs) or proteoglycans (PGs) and by gap junctions [128,134].

Bone has a regenerative capacity of its own and can heal without scarring in case of an uneventful healing. However, in cases with large or critical sized defects, it requires additional support and stabilisation for healing and regeneration. Hard tissue defect’s aetiology in the orofacial region can be attributed to genetic or congenital malformations, trauma, infections, cancer, and several other systemic or local pathologies. In oral surgery, its application extends from minor defects like periodontal pockets to moderate bone abnormalities like maxillary sinus lift and to much larger bone defects like mandibular and craniofacial reconstruction. Given the demand, bone regeneration is of primary importance in this field and bone is the second most transplanted tissue after blood. Extensive preclinical research has been done in this regard, with only a few therapies making it to the clinics [136]. Thus, it is highly imperative to look for the most efficient and beneficial ways of bone regeneration while ensuring the integrity and maintenance of the surrounding tissues and its functions. In this section, we will discuss the ideal requirements for regenerative therapies that will ensure highest biological function of the regenerated tissue, highlighting the past and current developments in the field.

#### 3.1.2. Determinants of Biomimetics for Bone Regeneration

The prerequisite for biomimetic regenerative therapies is having complete knowledge of the tissue to be worked with. Given the high complexity and multifunctionality of bone, the standard for regenerative therapies is set high. The biologically active agents that aid in bone regeneration should ensure adhesion, migration, proliferation, and differentiation of osteoprogenitor cells, thus resulting in an acceptable osseointegration. Moreover, considerations for optimal ECM and blood vessel formation are required for a satisfactory result.

(A) Mechanical and compressive strength: Human cortical bone and cancellous bone compressive strength ranges from 90–230 MPa and 2–45 MPa, respectively [137]. The compressive and tensile strength, and the density and fracture toughness of the graft should be comparable to that of the recipient site. Moreover, it has been shown that the scaffolds’ stiffness can have a direct effect on the behaviour of the surrounding cells [138]. Therefore, it becomes critical to choose the material according to the site of procedure and the desired outcome.

(B) Surface properties: Surface differences, even at nanoscale ranges, can affect the behaviour of cells. They have a regulatory effect over several osteoblastic functions like cell adhesion, migration, proliferation, cell signalling, genetic expression, and stem cell fate. It is attributed to properties like increased surface area and roughness; increased wettability and porosity, which in turn increases nutrient exchange; and protein absorption [139].

(C) Pore size: It is a critical determinant in bone regeneration and repair as it allows for easy exchange of bone and blood cells, along with other nutrients within the bone. Natural grafts have the advantage of inherently possessing the ideal pore size, while several parameters are considered in fabricating synthetic graft materials. The pore size recommended previously was 0.3 mm to 0.5 mm to allow proper vascularization and osteogenesis. Pamula et al. used a poly-L-lactide-co-glycolide (PLG) scaffold with equal pore density but different pore size. Biocompatibility was measured by comparing the penetration of osteoblast-like cells and the expression of bone reforming proteins like osteopontin and osteocalcin. The growth and penetration were seen more with pore size 0.4 mm to 0.6 mm, indicating that a larger pore size than recommended before is favourable for osteogenesis [140]. Ghayor et al. demonstrated that material with a pore size of the range 0.7 to 1.2 mm performed better in in vivo models for calvarial defects [141].

(D) Controlled biodegradability and dimensional stability: Most commonly, the grafts should be absorbed and replaced by natural tissue over time. Therefore, it requires the material to have intermediate biodegradability, which corresponds to the simultaneously ongoing natural remodelling process [142]. If the graft resorbs prematurely, there is a possibility of graft collapse within the defect and eventually failed restoration. On the other hand, delayed resorption can interfere with natural bone deposition, elicit immunogenic reactions, and thus decrease biocompatibility. In general, a decrease in particle size and an increase in porosity of biomaterials reduce the mechanical strength and enhance the biodegradation rate. Also, nanomaterials undergo faster and more homogenous biodegradation than conventional micron-based materials. Predictably, biphasic and composite materials exhibit degradation rates intermediate between the two depending on the percentage of each phase. For example, Hydroxyapatite (HA)-based materials have a very low degradation rate, while tri-calcium phosphate (TCP) and organic grafts have higher. Thus, biphasic (HA + TCP) grafts have to be used with special consideration for the type of recipient site and its degradation profile [143]. Good dimensional stability allows for chairside adaptation of the bone graft to the defect.

(E) Biocompatibility: For a graft to be biocompatible, it should allow growth, differentiation, and attachment of osteogenic cells and have antimicrobial and appropriate inflammatory properties. Inflammatory responses to some extent aid in the integration of the graft or implant material as it leads to remodelling of the tissues by enhancing angiogenesis, by removing debris generated during the surgical procedure, and by enhancing chemotaxis of reparative cells including pluri-/multipotent cells [144]. Some materials need to get resorbed by the natural host response mechanisms, while others which have to be implanted for longer terms require degradation just enough to ensure high tissue integration. Thus, different levels and extent of inflammatory response are desired depending on the purpose of the regenerative procedure [145]. Furthermore, in Anthony Gristina’s language, successful osseointegration is a “race for the surface” between microbial colonization and tissue cell integration [146]. Harnessing the antimicrobial response is critical to ensure successful graft, further discussed in the following periodontology section.

#### 3.1.3. Bone Grafts and Scaffolds

(A) Natural grafts

(i) Autograft: It is a part of the patients’ own tissue and has been considered the gold standard for bone defect repair for years [147,148]. They are commonly obtained from the iliac crest, parietal bone, and mandible. However, it becomes critical to have a second surgical procedure at the tissue harvest site, which increases the risk of developing morbid complications such as donor site injury, deformity, and scarring. It is also associated with higher costs for the surgical procedure, longer recovery times, excessive bleeding, pain, inflammation, and occasionally infections. However, they have a limited role in restoration of larger defects, which requires a higher amount of bone. The issues mentioned above have made other graft materials as a more lucrative alternative in clinical practice [149].

Another exciting autologous bone graft procedure is by ectopic prefabrication, where heterotopic ossification is achieved to produce bone tissue by placing a scaffold in vivo. It utilises the principle of Wolf’s law, according to which muscles and bones mechanically and functionally perform together and have an organizational effect upon one another [150]. Wang et al. successfully demonstrated the fabrication of mandibular bony construct by placing a mandible-shaped titanium mesh scaffold packed with cancellous autologous blocks from dog ribs into the latissimus dorsi muscle with thoracodorsal artery and vein through the scaffold [151]. Similar studies have been done by Kokemüller et al. (2014), Naujokat et al. (2019), and Warnke et al. (2004). This method of bone regeneration seems promising, with an excellent review by Huang et al. [152].

(ii) Allografts include grafts taken from living donors or cadavers and are the second most commonly used bone grafts [153]. They offer similar advantages as autografts and can be harvested as various bone matrices: cancellous chips, cortico-cancellous grafts, cortical grafts, osteochondral, and whole bone segments. However, they have a higher risk of developing immune reactions due to mismatched genetics. Additionally, there is a risk of transmission of infection. Therefore, the grafts are devitalised through decalcification, deproteinization, irradiation, and freeze-drying procedures for preservation. The aforementioned processing makes the osteoinductive potential negligible as the cells are lost. Blume et al. recently used an allograft to fabricate a customized bone graft using CAD/CAM technology which fit the large defect perfectly and thus was a clinical success [154].

(iii) Xenografts: Xenografts are any graft across different species. They offer similar advantages and drawbacks like the allografts, but the immunogenicity is expected to be higher along with higher risk of cross-species infection. Interestingly, the phenomenon is seen to be lesser with these grafts, and thus, they have emerged as promising candidates as transplant material [155,156]. Decellularized Bone (DCB) remains the most common graft material. Recently, growth factor-enhanced DCB has been developed. Also, the addition of doxycycline to Bovine Hydroxyapatite (BHA) was observed to be more stable than the graft without it, due to its nonantibiotic effects on fibroblasts, mesenchymal cells, and osteogenic cells, which promote cell adhesion and, ultimately, cell proliferation and differentiation [157]. Notable FDA-approved DCB products include Graftech^®^, GraftCage^®^, BTB Select^®^, BioCAP Select™, MatriGRAFT^®^, and ReadiGRAFT^®^ [158]. A summary of all the natural grafts is given in Table 6.

(B) Natural polymers.

A diverse group of naturally occurring substances represent the constitution of the extracellular matrix (ECM). Thus, they can be used as scaffolds for bone regeneration with or without the combination of stem cells and growth factors, more commonly employed in a technique called guided bone regeneration (GBR). A few of the polymers, as described by Haugen et al., can be classified as proteins (collagen, gelatin, and fibrinogen, elastin); polysaccharides (glycosaminoglycans, cellulose, and amylose); and polynucleotides (DNA and RNA) [136].

(i) Autologous ECM-based substitutes: Comparable performance of decellularized allogeneic and xenogeneic bone grafts to autogenous bone grafts indicates the importance of ECM in bone. Paduano et al. examined in vitro osteogenic induction of dental pulp stem cells (DPSCs) by culturing them on decellularized bone matrix (bECM) as well as collagen type 1 matrix (Col-1). bECM with osteogenic growth factors showed maximum expression of osteo-specific markers as compared to the Col-1 matrix [159].

(ii) Proteins: Collagen, elastin, fibrinogen are some of the abundant extracellular matrix proteins. Scaffolds derived from these can be manufactured to simulate natural ECM and, when used with integration of growth factors or living cells, can offer osteoconductive and osteoinductive properties [160]. More recently, bioinspired proteins have gained attention as biomimetic scaffolds for hard tissue regeneration [161].

(iii) Marine products: They offer a possible source of bone substitutes as they can mimic bone with their biochemical composition, structural arrangement, and biofouling ability [162]. Marine products include collagens from jellyfish, polymers from marine diatoms, chitin from marine sponges, and hydroxyapatite and calcium phosphates from fishbone and other organisms. [163,164] Sensing the osteogenic potential of marine products, Pinctada’s powdered nacre was used for maxillary augmentation by Atlan et al. as early as 1990, while recently, Coringa et al. studied bone substitutes from oysters in mandibular defects in an animal model [165]. Advances have been made for chemical modification of marine products and their use as scaffolds for pluripotent cells’ culture [166]. Chitosan, a polymer initially derived from marine organisms, is now widely studied for bone regeneration [167,168].

(C) Synthetic graft materials

(i) Bioceramics:

Ceramic scaffolds are derived from bioactive inorganic materials. They offer the advantage of providing a similar biochemical composition of the inorganic phase of natural bone tissue. Most commonly, Calcium phosphate (CaPs)-derived scaffolds are used, amongst which most popular are hydroxyapatite (HA) and tricalcium phosphate (TCP). CaPs have the ability for osteoconduction by allowing osteoblasts to attach, proliferate, and differentiate [169]. On several occasions, CaPs have been seen to have osteoinductive effects as well [170]. This is attributed to the topography, porosity (both size and saturation), and composition of these materials, which are believed to allow adsorption, entrapment, and the final concentration of circulating osteogenic factors and osteoprogenitor cells. HA and TCP are usually used as biphasic composites (BCP). HA is insoluble and thus maintains the space and structure, while TCP stimulates new bone formation by the dissolution of calcium and phosphate ions. Nevins et al. tested different compositions of BCP for alveolar ridge modification, where similar results were obtained for all, with the only difference being graft resorption. It was delayed by increasing the amount of HA, which is expected [171]. Janssen et al. used microstructured TCP in glycerol matrix in cleft palate repair as an alternative to autologous grafts and found them satisfactory [172]. It can be further highlighted that the origin and method of synthesis of these scaffolds can have a direct impact on the cellular responses. Marinucci et al. reported higher osteogenic induction with bovine-derived HA than HA alone when the genetic profiles of mesenchymal stem cells were compared [173]. Similar comparative studies have been done, which indicate that, even if the broader outcomes seem similar, there are differences in minute cellular responses which might cause differences in the regenerative potential and effect of a graft [174]. This variability can be attributed to the difference in their dissolution/precipitation behaviour, microporosity, physicochemical properties, surface area, and topography [175]. Thus, the graft materials have to be thoroughly studied and carefully selected for the intended purpose.

Bioactive glasses that contain calcium can produce a bioactive hydroxy carbonated apatite layer in biological fluids that can be biologically integrated into the tissues [176]. Further, their resorption rates can be customized to make it possible to release bioactive molecules at a controlled rate. Although the brittleness of these materials is a concern, efforts are being made to produce a scaffold of comparable mechanical properties to that of bone [177].

(ii) Synthetic polymers: For bone regeneration, the most commonly used polymers are aliphatic polyesters poly-lactic acid (PLA), poly-lactide-co-gylcolide (PGLA), poly-ε-caprolactone (PCL), polyhydroxyalkanoates (PHA), polyglycerol sebacate (PGS), and poly-glycolic acid (PGA) [178]. They have tuneable biomechanical, biodegradability, and structural properties. Nevertheless, they have limited osteoconduction as they have low cell attachment capacity [178]. Also, they are mechanically stiff, which contradicts the flexibility of natural bone. To overcome the brittleness of bio-ceramics and stiffness of polymers, composite polymers have been widely explored [179]. Collagen-HA was considered as a promising candidate. Eventually, biomimetic mineralised collagen (MC) was introduced [180]. Feng et al. showed that MC gave better clinical outcomes for socket preservation than collagen-HA [181].

(D) Applications and advances

(i) Micro-/nanofibres and nanoparticles

These are produced through electrospinning, which enables the manufacture of extracellular matrix (ECM)-like structures. They have high porosity, specific surface area, and nano-topography, modulating cellular behaviour to promote cell adhesion, proliferation, and differentiation by mimicking the ECM. Several recent studies have reported the differentiation of Mesenchymal stem cells (MSCs) and other multipotent cells into mineralising osteoblasts when cultured on electrospun nanofibres [182,183]. Electrospun PCL scaffold was used by Puwanun et al. to assess the differentiation of mesenchymal stem cells from jaw and periosteal tissue [184]. Although clinical studies with nanofibres in dentistry are still emerging, one of the clinical trials with nanohydroxyapatite was done by Lombardi et al. Mean sinus pneumatization was significantly lesser (*p* = 0.15), and crestal bone resorption was less as well (*p* = 0.24), indicating the potential role of these therapies in socket preservation after molar, especially third molar extraction [185].

(ii) Nanocomposites

Nanoscale features have a regulatory effect over profuse osteoblastic cellular functions like cell adhesion, migration, proliferation, cell signalling, genetic expression, and stem cell fate. Zhang et al. reported the nanohydroxyapatite’s compressive strength (poly-l-lactic acid nanocomposites), which was greatly enhanced and reached 115 MPa, comparable to natural bone [186]. Nanohydroxyapatite-covered polyhydroxy butyrate (PHB) fibres obtained through electrospinning showed better results than simple PHB scaffolds [187]. Biocompatible nanocomposite with polyurethane, chitosan, and TCA was fabricated and loaded with amoxicillin, which offers a promising approach for bone tissue engineering [186,188].

Similarly, drug loading can be enhanced using nanocomposites to elicit drug delivery, most commonly needed for anti-inflammatory and antibiotic effects. Recently, Shi et al. fabricated a novel twin-fibre membrane with antibacterial and osteoinductive properties. The drug’s slow release matched with natural bone regeneration, and the material proved to be biocompatible and had improved osteogenic properties. It can serve as biomimetic multifunctional artificial periosteum, the natural layer responsible for bone regeneration [189]. Similarly, a composite scaffold with PLA, PCL blended with nanoHA, and cefixime complex was synthesized by Sharif et al. with potential application in oral and maxillofacial related therapies [190].

In conclusion, most widely used bone substitutes in dentistry are still naturally derived grafts and natural polymers in combination with bio-ceramics. Several recent clinical trials are listed in Table 7. In a metanalysis study of bone graft substitutes, Corbella et al. found that autogenous bone (AB) alone leads to significantly higher osteogenesis in comparison to Bovine bone (BB) (*p* = 0.04) In contrast, no significant difference was found when BB was compared with a mixture of AB and BB (*p* = 0.52) [191]. Moreover, BB showed higher bone formation than HA alone (*p* < 0.001), but a mixture of HA with TCP showed better results than BB (*p* < 0.001) [191]. In another metanalysis, Jensen et al. showed that synthetic bone grafts showed significantly less clinical outcomes [192]. Thus, natural and biologically closer substitutes offer higher clinical success [192].

#### 3.1.4. Cell Therapy

(A) Stem cells

Cells, either tissue-specific or multipotent, have an immense role in tissue engineering research, both in vivo and in vitro, and for treatment as well as testing for different grafts/scaffolds. Variations in the studies call for standardized cell models that can be reproducible in all the settings. Palumbo et al. compared the behaviour of committed human osteoblast cells from bone biopsies with multipotent human dental pulp cells (hDPSC) from extracted teeth to identify cellular models for bone regeneration. They found that committed osteoblast cells are useful for identifying and testing materials and surfaces osseointegration while hDPSCs are more useful for obtaining in vitro osteocyte-like network for bone regeneration [199]. Having established the cell models, there have been numerous studies regarding cell therapies. For bone regeneration, a wide range of pluripotent stem cells have been studied in oral and maxillofacial surgery, human umbilical cord mesenchymal stem cells [200], bone marrow-derived stem cells [201], adipose-derived stem cells [202], mesenchymal dental pulp stem cells [203,204], periodontal ligament stem cells [205], and SHED [206]. While the wide use of these cells seems promising, strict vigilance and regulation is required to ensure standardized treatment outcomes. Given the unpredictable nature of multipotent and pluripotent cells, stem cell-derived conditioned media are getting more attention [199,207,208]. 

(B) Hybrid scaffolds

CaP-based scaffolds, as discussed above, are osteoinductive due to their nurturing nature towards the cells and accommodation for growth factors. To potentiate the osteoinductive capacity, scaffolds integrated with osteoprogenitor cells and factors were developed [204,209,210]. Korn et al. studied substitutes for autologous grafts in rodent models for alveolar cleft alveoloplasty using bHA alone, bHA with undifferentiated MSC, and bHA with osteogenically differentiated MSCs, where the last group showed the best bone growth [211]. Strontium folate (SrFO) derivatives were integrated into biohybrid scaffolds given their role in treating osteoporosis and other bone diseases. Martin-del-Campo et al. loaded the SrFO derivatives in TCP and chitosan polyethylene dimethacrylate scaffolds, which were then seeded with DPSCs. They observed significantly improved results with SrFO-integrated scaffolds in terms of bone formation [212]. In a clinical study by Al Ahmady et al., autologous bone marrow mononuclear cells combined with platelet-rich fibrin and nanohydroxyapatite were used to treat alveolar cleft. It led to lesser donor site complications, faster soft tissue healing, and less postoperative pain [213].

#### 3.1.5. Cell-Free Therapies

**Figure 5:**
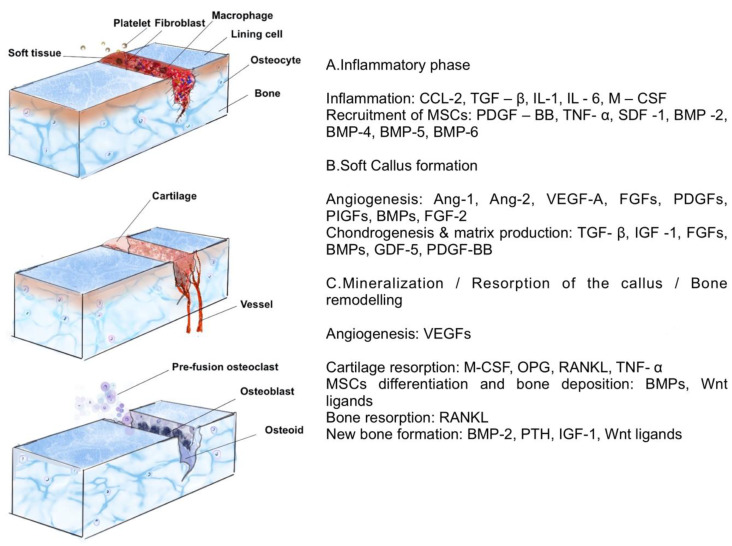
The temporal progression of fracture healing: healing of a fracture involves a complex series of processes which can be broadly divided into four phases, A. inflammatory phase; B. soft callus formation, C. mineralisation of callus, and bone remodelling. Each phase is regulated by key growth factors, as shown in the figure. BMP = bone morphogenetic protein, FGF = fibroblast growth factor, GDF-5 = growth/differentiation factor 5, IGF-1 = insulin-like growth factor 1, M-CSF = macrophage colony-stimulating factor, OPG = osteoprotegerin, PDGF = platelet-derived growth factor, PlGF = placental growth factor, PTH = parathyroid hormone, RANKL = receptor activator of nuclear factor κB ligand, SDF-1 = stromal cell-derived factor 1, TGF-β = transforming growth factor β, TNF-α = tumor necrosis factor α, and VEGF = vascular endothelial growth factor. Image adapted from Yague et al. [216].

Bone healing is a complex and highly coordinated process, generally divided into inflammation, renewal, and remodelling (Figure 5). Mimicking this process by using the appropriate growth factors and small molecules in a regenerative field can help achieve a successful clinical outcome [214]. Furthermore, these growth factors are also responsible for the recruitment and maturation of osteoprogenitor cells, especially mesenchymal stem cells. Therefore, they can perform on-site programming of the cells [215].

(A) Bone morphogenetic protein (BMP)

It has emerged as the most promising medium for bone regeneration [217,218]. Sudheesh et al. studied a biphasic polycaprolactone construct combined with hyaluronic acid-based hydrogel and loaded with BMP-2 for correction of vertical bone height in the mandible in rabbits. The outer wall of the biphasic material simulated cortical bone, while the core simulated medullary bone. BMP was released in a sustained manner from the construct, while it provided mechanical and space maintenance properties through the cortical part and osteogenesis and angiogenesis through the medullary [219]. Gene delivery is also a lucrative method as it ensures prolonged and stable production of the protein, reviewed by Park et al. for BMP2 in dentistry [220]. In a meta-analysis for animal studies for gene delivery in maxillofacial bone defects, Fliefel et al. found gene delivery as better therapeutics [221].

(B) The vascular endothelial-derived growth factor (VEGF), and IGF1 and 2

The VEGF pathway is considered critical for angiogenesis, which indirectly affects osteogenesis [222]. VEGF acts synergistically with BMP and other growth factors [223]. Kim et al. used FGF with BMP2 to show improved maxillary alveolar bone regeneration [224]. FGF in low concentration is seen to improve osteogenesis. Kitamura et al. tested its clinical potential in an RCT and successfully treated patients with periodontal defects [225]. IGF 1 and 2 have roles in osteoblast differentiation, stimulation of bone matrix deposition, and collagenous and non-collagenous protein expression and thus was tested for osteogenic differentiation of MSCs by Reible et al. [226].

(C) Platelet-derived growth factor (PGDF)

PGDF promotes osteogenesis as well as angiogenesis and has been shown to be successful in periodontal tissue regeneration [227]. In a systematic review by Li et al., BMP was concluded to be less effective than PDGF [228]. A rich source of growth factors is platelet-derived products like Platelet-rich plasma (PRP) and Platelet-rich fibrin (PRF). PRP is recommended for faster delivery and PRF for the stable delivery of factors [229]. Recently, Stumbras et al. performed alveolar ridge preservation using bone substitutes and autologous platelet concentrate, where they found that plasma-rich growth factors perform better than the grafts [230]. Concentrated growth factor (CGF) has also emerged as an excellent tool for oral regenerative medicine [231,232].

### 3.2. Biomimetics in Mucosal Repair

#### 3.2.1. Oral Mucosa

Oral mucosa is one of the most forgiving tissues in our body, which heals rapidly and without scarring, usually in smaller defects. It can be attributed to increased vascularity and faster turnover rates. Oral mucosa is a highly specialised tissue which is divided into alveolar and masticatory mucosa, depending on the site and function. Their healing becomes challenging in case of large defects where open wounds remain susceptible to infection and wound contracture. In these conditions, grafts are required to cover up the wound site to make up for the lost tissue and to act as barriers to ensure uneventful healing by preventing foreign body entrapment (dead cells and debris) and infection. Grafts for oral mucosal repair become challenging as it is constantly under wet environment due to oral fluids and is introduced to load due to mastication and food.

#### 3.2.2. Determinants of Biomimetics

Regenerated tissues are inevitably required to mimic the complex organization of mucosal layers to have an uneventful and successful wound healing. The desired outcomes for successful biomimicry are organization into multiple functional layers with ECM organization, maintenance of functionality, exhibition of volume stability, creation of an epithelial barrier, biocompatibility, lack of toxicity, and immunological rejection [233,234]. The regenerated tissues are inevitably required to mimic the complex organization of the mucosal layers to have uneventful and successful wound healing. 

#### 3.2.3. Mucosal Grafts

**Figure 6:**
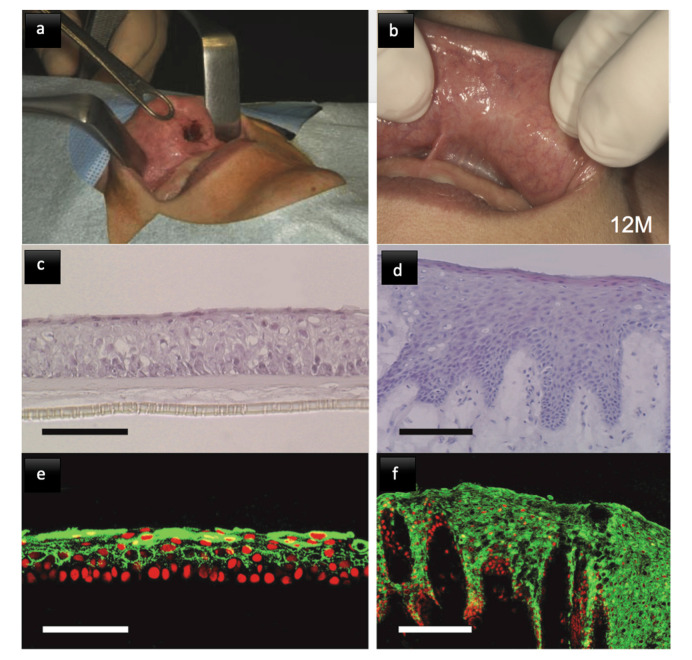
(**a**,**b**) Auto transplantation procedure for an oral mucosal defect for pleomorphic adenoma at the time of surgery and 12 months after surgery, respectively; (**c–f**) morphology and keratin expression patterns of amniotic membrane-cultured oral mucosal cells and oral mucosa; haematoxylin and eosin stained mucosal epithelial cells exhibiting seven differentiated and stratified layers (**c**) as compared to oral mucosa in vivo (**d**); keratins (green) expressed in the cultured mucosal cells (**e**) vs. oral mucosa (**f**); and nuclei stained with propidium iodide (red). Scale bars: (**c**,**e**) 100 μm and (**d**,**f**) 200 μm. Images derived from Amemiya et al. [236].

(A) Autologous grafts till today remain the gold standard. Conventional techniques that are still most widely used include local flaps, distal flaps, and free vascularized flaps from a donor site. However, their limitation in large defects and donor site morbidity leads to the search for new regenerative scaffolds and tissues. Cell culturing techniques for autologous cell sheet formation offer advantages of an autologous graft without the donor site’s involvement. Keratinocyte-based products are the ex vivo produced oral mucosal equivalent (EVPOME), which are constructed by cultivation of autogenous keratinocytes [235]. Amemiya et al. have done tremendous work over the years to develop autologous graft cultures over the human amniotic membrane, with a successful clinical trial done recently (Figure 6) [236]. Fibroblast-based constructs involve culture of embryonic fibroblasts or autologous fibroblasts, which are cultures over a collagen matrix. The scaffold resorbs upon transplantation, leaving the ECM and functional fibroblasts which secrete appropriate growth factors, allowing faster healing of the tissue [237].

(B) Natural polymers and scaffolds

Human-derived Acellular Dermal Matrices (ADM) are one of the more common scaffold materials which mimic ECM to provide a favourable environment for cell growth and growth factor exchange [238]. Similar scaffolds can be generated of xenogeneic origin like porcine graft, which showed positive outcomes as a scaffold [239]. Collagen and chitin fibres are a few of the other natural polymers which can be used in various forms, microscopic to nanometric, to form scaffolds for cell proliferation [240]. A comprehensive review of synthetic and natural graft materials has been provided by Toledano et al. [241].

(C) Synthetic polymers

The most common synthetic polymers used as mentioned above as well are poly(ε-caprolactone) (PCL), poly (glycolic acid) (PGA), poly (lactic acid) (PLA), poly (hydroxyl butyric acid), and poly (hydroxyl valeric acid), all of which are resorbable. They offer more significant advantages over natural ones, as they have longer shelf life, can be manufactured in bulk, and have better control over physical and chemical properties. However, their stiffness and fragility were seen to increase because they have been recently used mixed with non-resorbable polymers, (MMA)_1_-co-(HEMA)_1_ and (MA)_3_-co-(HEA)_2._ As the synthetic grafts have limited biological interactions, they have to be impregnated with bioactive molecules, which can be growth factors directly (BMPs and VEGF) or other molecules with stimulatory effects like Si and zinc oxide [242]. Electrospun nanofibres, as mentioned before, offer greater surface area and favourable porosity in addition to the capacity to carry growth factors as well as drugs such as antibiotics. They can be used as a dressing in relieving patient discomfort in patients with oral mucosal defects [243]. The upcoming strategies for mucosal regeneration have outstanding clinical outcomes, are economically sound, and can be harvested in greater quantities, thus providing reliable alternatives to conventional autografts.

## 4. Biomimetics of Periodontal Tissue Engineering and Regeneration

Periodontium is another highly specialised tissue in the oral and maxillofacial region. Common aetiologies for its loss are periodontitis and loss of tooth due to trauma or caries. Periodontitis is an inflammatory disease that leads to destruction of the tooth attachment apparatus, consisting of the gingival tissue, alveolar bone, cementum, and periodontal ligament (PDL). It is also associated with several systemic diseases such as cardiovascular diseases and rheumatoid arthritis. It is one of the most widespread infectious diseases and is the leading cause of tooth loss in adults [244]. Periodontal treatment aims to arrest further progression of the disease and to restore tissue integrity. Scaling and root planing are nonsurgical mechanical approaches to control disease progression [245]. Once tissue destruction has occurred, regenerative treatments are needed. Here, we have highlighted the key strategies in periodontal tissue regeneration, with a brief view on biomimetic strategies for oral implantology.

### 4.1. Periodontal Regeneration

#### 4.1.1. Cell-Based Therapies

Currently, there are several surgical techniques used for periodontal regenerative therapy, including guided tissue regeneration (GTR), bone graft placement, as well as a variety of different biomaterials and growth factors [246]. Each technique has its advantages, yet the ability to completely regenerate the damaged periodontal structures has not been achieved in patients, especially in those with advanced periodontal defects [247]. In recent years, progress in stem cell biology and tissue engineering has ushered in stem cell-based approaches in regenerative therapies. Many studies have shown that stem cells can be used in conjunction with different physical matrices to regenerate periodontal tissues in vivo [248,249,250]. Stem cells used for periodontal therapy are usually mesenchymal stem cells (MSCs), since these cells are capable of differentiating into many tissues (bone, ligament, muscle, etc.) [251]. Also, they have an immunosuppressive capacity that gives them essential features in autologous and allogenic transplantation [252]. MSCs can be isolated from a variety of sources. Studies using animal models have shown successful periodontal tissue regeneration with MSCs derived from bone marrow [253], adipose tissue [254], and PDL [255]. While there is no consensus on which tissue source provides the most appropriate stem cells, researchers have focused on stem cells obtained from the oral cavity due to their accessibility as well as their high differentiation and proliferation abilities [256]. Oral stem cells have neural crest origin and thus represent a transient population of embryonic pluripotent stem cells [257]. Induced pluripotent stem (iPS) cells and embryonic stem cells have also been proposed for periodontal regeneration [258]. However, there seem to be limitations to this and it has not been carried out in practice [259]. There is evidence that the cells of PDL tissues are able to form a complete periodontal attachment apparatus [248]. One study examined the immunomodulatory properties of PDL stem cells, a type of MSC, and found that PDL stem cells inhibited the proliferation of activated peripheral blood mononuclear cells (PMBCs) via a partly dependent mechanism through interferon-γ, which is synthesized by activated PMBCs [258,260]. The disadvantage of PDL stem cells is that the cell regenerative potential seems to decrease as a function of the age of the donor [261]. Traditionally, PDL cells were collected from extracted teeth, which presented an issue because of the limited amount of PDL tissue on extracted teeth [255]. This is because stem cells are required in large quantities to accomplish clinical trials [254]. However, recent studies have shown that PDL stem cells can be procured from inflamed tissue within a periodontal defect [262]. This provides an easy and rapid alternative method to obtain PDL stem cells without extracting teeth.

Cells implanted into periodontal defects in immunocompromised rodents can regenerate cementum and PDL-like structures and can support periodontal tissue repair [263]. Other similar studies have demonstrated cementum, new bone, and PDL formation in larger animals like dogs [264] and pigs [265]. One study evaluated the regeneration of peri-implantitis defects in dogs using genetically modified PDL stem cells. Bone morphogenetic proteins (BMPs) have great potential in the regeneration of periodontal tissues [266]. Following the ex vivo transfer of the BMP-2 gene into PDL stem cells using an adenoviral vector, modified stem cells were implanted into the defects. This resulted in enhanced new bone formation and re-osseointegration in peri-implantitis defects compared with direct BMP administration to periodontal lesions [267]. Another finding that demonstrates the PDL cells’ ability for bone regeneration and maintenance is that they release important humoral factors to maintain alveolar bone. When PDL stem cells are cultured in an indirect co-culture model, they inhibit osteoclastic activity of alveolar bone-derived stromal cells, therefore preserving alveolar bone [268]. Biomaterials used in combination with stem cells have the potential to improve the beneficial effects shown by cell therapy and lead to better control of cell delivery to the target site and to decrease the number of cells lost. They also play an essential role in the delivery of biological agents that enhance interaction with the host tissue and improve the cell differentiation process [269]. Because there are several different biomaterials available (natural biomaterials, ceramic biomaterials, and synthetic polymers), many animal studies have been conducted using different combinations of biomaterials and PDL stem cells for periodontal regeneration [270]. In one study, Tsunmanuma et al. showed that canine PDL cell sheets combined with a mixture of collagen and beta-tricalcium phosphate lead to improved cementum and PDL fibre formation in a canine 1-wall defect model [271]. Biological agents also have an effect on PDL cells in periodontal regenerating. For example, insulin-like growth factor-1 (IGF-1) has been shown to increase osteogenic differentiation and produces a cascade of downstream reactions, playing a pivotal role in cell-based periodontal tissue regeneration [272].

Over the past few years, stem cell-based periodontal therapies have begun testing in clinical settings [273,274]. In a 2016 RCT study by Chen et al., autologous PDL stem cells from extracted wisdom teeth were used as an adjuvant to graft materials (bovine-derived bone mineral materials) in GTR therapy to treat periodontal intrabony defects. While there was a significant decrease in bone defect, no statistically significant differences were detected between the PDL stem cell group and the control group [275]. However, the study demonstrated that it is safe to use PDL stem cells in treating periodontal intrabony defects in humans. Stem cell-based regenerative periodontal therapy is a promising new field that has the potential to prevent tooth loss, to avoid costly treatments, and to provide more effective and less invasive treatment options. While there remain many issues that need to be addressed before stem cell therapy becomes widely available, clinicians should continue to monitor these technologies’ progression.

#### 4.1.2. Cell-Free Therapies

Alveolar ridge deficiencies can complicate implant placement as well as other prosthodontic reconstruction. Therefore, to gain adequate alveolar ridge dimensions, many bone regeneration techniques have been used with conjunctive therapy to optimize bone growth. Specifically, Growth Factors (GFs) and Platelet-Rich Fibrin (PRF) techniques have been shown to aid in bone healing following graft placements. GFs are expressed during tissue healing and can promote tissue regeneration when used in surgical procedures [276]. PRF allows us to obtain GFs directly from the plasma and is therefore widely used to aid tissue regeneration. It has a positive effect on cell proliferation, migration, adhesion, and differentiation. Additionally, Strauss et al. have shown the anti-inflammatory properties of PRF [277], which support their role in wound healing and bone regeneration. According to a systematic review by Strauss et al., PRF does have a clinical benefit on ridge preservation and in the early phase of osseointegration. However, pain and soft tissue healing outcomes remain unclear [277]. Another systematic review by Dragonas et al. reported some benefit on soft tissue healing and post-op and swelling [278]. PRF used in conjugation with other bone grafts has shown better clinical outcomes than when used alone [279,280,281,282]. In patients with chronic periodontitis, the use of 1% metformin and PRF showed more pocket depth reduction and relative attachment level than either group alone [283]. Along with open-flap debridement, PRF has been shown to increase canine periodontitis treatment outcomes in animal studies [284].

Furthermore, growth factors alone or derived from cells have also given promising results, given the fact that endocrinal secretions of the cells are responsible for the regenerative effects in tissues [285]. A sequential application of basic fibroblast growth factors and BMP-2 synergistically promoted differentiation of periodontal ligament cells, suggesting their potential use for periodontal regeneration [286]. A meta-analysis, however, suggested that recombinant human BMP2 and PDGF-BB, in its current concentrations, did not induce a significant effect on tooth extraction socket healing, sinus augmentation, or reconstruction of alveolar defects. However, 0.3 mg/mL rhPDGF-BB may promote the healing of sockets [228]. Another meta-analysis showed that 0.3% rhFGF2 and 0.3 mg/mL rhPDGF-BB showed more periodontal regeneration capacity than other concentrations and control groups [287]. It was also shown that rhBMP-2 substantially increased bone levels in localised alveolar ridge augmentation procedures [288].

Apart from the growth factors, other proteins like amelogenin have an eminent role in periodontal regeneration, in addition to the enamel formation discussed above [289]. Specifically, Emdogain, a porcine-derived extract of enamel matrix with proteins like amelogenin has gained attention due to its proliferative effect on cementoblasts [290], osteoblasts [291], endothelial cells [291], gingival fibroblasts [291,292], and periodontal stem cells [293]; to gingival and periodontal fibre growth and attachment; and to an anti-inflammatory effect. A systematic review conducted by Esposito et al. found that adjunctive use of Emdogain regenerates around 1 mm more tissue than techniques like GTR alone. Moreover, Emdogain is simpler to use and has less complications [294]. No firm conclusion was drawn in other preclinical [295] and clinical systematic reviews, which can be attributed to the disparate nature of the studies [296].

Although PRF has been shown to have good tissue healing properties as it can be used like a guided tissue regeneration membrane, one of its main disadvantages is that it resorbs within seven days [297]. Comparatively, other membranes for periodontal regeneration typically require 4–6 weeks [298]. Overall, PRF and GFs have been shown to have significant clinical therapeutic outcomes when used in bone tissue regeneration. However, more research is necessary to assess their full clinical benefits and indications [297].

#### 4.1.3. Guided Tissue Regeneration

Guided Tissue Regeneration (GTR) is one of several procedures that has been developed to treat periodontitis. The biological principle of this procedure is on the basis that a membrane barrier can prevent undesirable types of tissue cells from migrating into a wound while simultaneously allowing desired cell types to repopulate the wound, therefore allowing the regeneration of the desired type of tissue [299]. GTR membranes are surgically implanted, and there are currently two types of materials used in these barriers in clinical research applications which are non-resorbable and resorbable materials [270]. Compared to non-resorbable ones, resorbable barriers are often favoured as they eliminate the necessity for a second surgical procedure to remove the barrier [300]. Non-resorbable materials used in GTRs include polytetrafluoroethylene (PTFE) or titanium [270], while resorbable materials are made of synthetic or natural polymers with different combinations of biomaterials that allow the development of membranes with various structural, chemical, and mechanical properties [270]. Common resorbable membranes on the market are based on either polyesters or tissue-derived collagen, which both have limitations including unpredictable degradation and weak mechanical properties [270,300]. The GTR membranes need to have the following characteristics: (1) biocompatibility to prevent inflammatory responses when interacting with host tissues, (2) a proper degradation profile that matches new tissue formation, (3) proper physical and mechanical characteristics for in vivo placement, and (4) enough sustained strength to adequately perform barrier function and circumvent membrane collapse. Considering these requirements, several research groups have been working to design membranes with predictable rates of degradation, structures to maintain mechanical properties and bioactive properties, such as calcium-phosphate based growth factors for bone formation [300]. Over the past decades, many different biomaterials and their combinations have been made and tested with various levels of success to regenerate destroyed periodontal tissues treated by GTR [301]. To illustrate, a research was aimed to engineer and regenerate human long bone tissue by creating scaffolds from nanoparticles to mimic the natural histological structure of human long bone. They focused on polymer nanoparticle compositions due to its superior mechanical properties, high durability, and surface bioactivity. The group concluded that they successfully produced degradable, bioactive, and permeable composite hollow fibre membranes with a wet phase phase-inversion approach for guided and biomimetic bone tissue engineering [302]. In another attempt to form membrane barrier designs for GTR that mimic naturally occurring biological processes, a study conducted by Zhang et al. used natural eggshell membrane, a semipermeable membrane with two unique layers [301]. The results from this study showed that the soluble eggshell protein with poly lactic-co acid nanofibre (SEP/PLGA) electrospun membrane they made formed an interconnected porous network with strong mechanical properties. Moreover, biological study results suggested that SEP/PLGA nanofibres have the potential to improve cell attachment, proliferation, and spreading. Therefore, the study showed the promising potential of SEP/PLGA nanofibres for future GTR membrane application [303]. Due to the limitations of current common barrier membranes used for GTR, many researchers have focused on developing improved barrier membranes, often with designs that mimic biological processes. Although recent studies have shown promising potential in the field of GTR, further advancements and research are required before they can be used to help patients [270].

### 4.2. Implant BIOMIMETICS

#### 4.2.1. Surface Modification and Alternative Materials for Implant Osseointegration

The most frequently used material in dental implantology is titanium; however, other materials such as zirconia have recently been introduced to the market [304]. Titanium is widely used due to its biocompatibility and mechanical properties, which allow for its osseointegration. An implant must be properly osseointegrated into the jaw to ensure long-term stability, resistance of biomechanical forces, and proper transfer of forces to the alveolar ridge, which in turn preserves bone [305]. Some of the drawbacks of titanium implants are its grey colour when placed in an aesthetic region in a patient with a thin biotype [306] and some rare yet reported titanium allergies [307]. The aesthetic disadvantage gave rise to the zirconium alternative, a bioinert non-resorbable metal oxide, which also shows comparable osseointegration [304]. A study comparing the peri-implant crevicular fluid (PICF) surrounding titanium and zirconium implants found no significant differences in the pro-inflammatory cytokine or bone metabolism mediators with the exception of leptin [308]. This finding correlates with the biocompatibility of both materials used in implantology. Zirconium implants are not as predictable as titanium implants. One study found degradation products of a zirconia-based implant only 29 months postoperatively [309]. Additional alternatives focusing on aesthetics include using a different system whereby the titanium implant’s external colouring is pink, which reduces the potential of soft tissues appearing grey while maintaining the predictability of a titanium implant [306].

Another way to improve the osseointegration of implants is through surface modification of the titanium and zirconium. A study compared the osseointegration of zirconium implants with their surface modified through either blasting, etching, or both methods simultaneously [304]. All three modifications resulted in good biocompatibility and osseointegration when compared to the reference zirconium implant. They also had a better attachment to the gingival and bone tissue around the neck area compared to the reference implant [304]. In addition, surface modification using platelet-rich plasma has also been investigated. Platelet-rich plasma (PRP) is used to deliver osteogenic and angiogenic growth factors to speed up bone regeneration and tissue repair [310]. It stimulates the proliferation and differentiation of mesenchymal cells to osteoblasts to help with bone healing [310]. The PRP used in surgery is prepared from the patient’s blood through centrifugation [311], and the implant’s surface is later treated with the PRP. A randomized controlled study found that the PRP application onto the implants’ surface enhanced stability and bone healing [312]. However, a recent systematic review investigating the use of PRP to aid in bone healing and implant success found that, while most studies had positive results, the predictability and effect that PRP application onto implants has on bone regeneration, osseointegration, and implant stability and success remains unclear [313].

#### 4.2.2. Antimicrobial/Anti-Inflammatory Aspects of Oral Implantology

The oral cavity utilises a plaque biofilm to shield itself against microorganisms with its constant contact with various pathogenic microorganisms. However, plaque biofilm is one of the contributing factors to caries and other various dental diseases, such as periodontitis. To tackle this issue, various studies have been investigating antimicrobial agents as a solution [314,315]. Unfortunately, results are not promising due to the decreased efficiency of antibacterial agents as they are released and degraded quickly [314,315].

To increase antibacterial agents’ efficiency, many studies have been investigating nanoparticles to ultimately minimize the development of dental diseases [314,316,317]. Nanoparticles carry unique physiochemical properties, including high charge density and large surface areas, which allow them to interact with the negatively charged surface of bacterial cells, preventing bacteria’s cellular functions [314,316,317]. Thus, these properties allow the nanoparticles to maintain high levels of antimicrobial activity. The potential of nanoparticles can further be applied to the field of periodontology. The combination of dimethylaminohexadecyl methacrylate (DMAHDM), 2-methacryloyloxyethyl phosphorylcholine (MPC), and the nanoparticles of amorphous calcium phosphate (NACP) has been shown to be highly effective against pathogens associated with periodontitis [314,318,319,320]. In the presence of an in vitro subgingival biofilm, nanoparticles with Ag^+^, Zn^2+^, doxycycline, or synergistic with chlorhexidine (CHX) have also demonstrated the ability to prevent development of pathogenic microorganisms [314,318,319,321]. Furthermore, the nanoparticles with Ag present in toothpastes have been shown to diminish the presence of pathogens associated with periodontic diseases [314,322,323]. Glass and glass-ceramics are viewed as another means of enhancing antibacterial activities in the oral cavity [324]. Such structures are developed commonly by silica, composed of Si^4+^, B^3+^, and P^3+^, and by the glass’s heat treatment [324,325,326,327,328,329,330]. In addition, these compositions play a crucial role in how practical their antibacterial activities are. With their unique ion-containing matrices, glass and glass-ceramics possess bioactive properties, including osteoinductive and osteoconductive abilities, allowing them to bond with bone tissues [324,331,332,333,334]. Glass and glass-ceramics have antibacterial activities as their surface matrices are integrated with ions, including Ag^+^, Ce^3+^, Cu^+^, Sr^2+^, and Zn^2+^ [324,331,335,336,337,338]. The rate at which these ions are released is directly correlated to the glass or glass-ceramic surfaces’ roughness level. The higher the surface roughness of the structure, the higher the rate at which the ions are released. Fernandes et al. noted that, among these ions, Ag^+^ is the common ion that is doped on glass and glass-ceramics studied in recent studies [339]. Hence, these materials play a crucial role at the site of dental implants as *Escherichia coli, Staphylococcus aureus*, *S. epidermidis*, and *Pseudomonas aeruginosa* are identified to be present in 90% of all dental implants [339,340,341,342]. It has been demonstrated that 45S5 Bioglass, which contains hydroxycarbonate apatite on its surface, can suppress *S. sanguis*, *S. mutans*, and *Actinomyces viscosus* [318,339]. A study by Fernandes et al. has shown that 45S5, which is a glass or glass-ceramics containing silicon, inhibits the development of *E. coli*, *P. aeruginosa*, *S. epidermidis*, *Moraxella catarrhalis*, and *Enterococcus faecalis* [339]. These examples of the glass structures indicate a means of delivering a significant antimicrobial activity in the oral cavity.

The use of titanium surface coating has been explored as a means to introduce antibacterial properties on implant settings as titanium is highly biocompatible and resistant against corrosion. Although titanium alloy alone is not effective to provide antibacterial properties, its combination with chitosan allows this co-polymer to prevent biofilm formation and bacterial growth on implant surfaces [343,344,345]. Chitosan is a linear polysaccharide that consists of *N*-acetyl-d-glucosamine and d-glucosamine units for which position 1 and 4 contain B links. Its unique structure provides chitosan with a range of properties, including its antibacterial ability, biodegradability, and cytocompatibility [345,346,347,348,349,350,351,352]. However, its variety of biological properties, especially its antibacterial properties, depends on the characteristic of chitosan, which includes origin, molar mass, the degree of acylation, and its condition at production. In addition, chitosan can be produced in various forms, such as nanoparticles, fibres, gels, membranes, and sponges, but each form has different effects on chitosan’s biological properties [345,353,354,355,356]. A study by D’Almeida et al. (2017) found that a combination of titanium alloy and non-animal chitosan, which was developed using the coupling agent triethoxysilylpropyl succinic anhydride (TESPSA), was effective against the growth of *E. coli* and *S. aureus* [345,357]. This combination represents as an ideal coating due to the biocompatibility of titanium surface and the use of non-animal chitosan, which make the combination as non-allergic and tolerable for the oral cavity [345]. Overall, nanoparticles, glass-ceramics-based materials, and chitosan-coated titanium represent effective means to hinder the activity of pathogenic microorganisms where dental implants are placed. There are various combinations of these materials, and further research on these materials will help determine the most effective one in clinical settings.

## 5. Conclusions

In this review, we emphasized the current scenario of biomimetic analogues used in dentistry. It is evident that intensive research over the years has led to the development of highly innovative, futuristic biomaterials, and techniques to simulate and replace natural structures in the craniofacial region. Nevertheless, as a biomimetic consideration, naturally derived or biologically close materials are noted to have better clinical outcomes with higher chances for clinical translation and patient use. This can be attributed to the multifarious nature of biological systems, which are an interplay of physiological, physiochemical, mechanical, and metabolic processes occurring simultaneously. Thus, there is a need for an interdisciplinary approach integrating medicine, bioengineering, biotechnology, and computational sciences to advance the current research in dentofacial regeneration. A wide range of in vitro and animal model studies prove that novel treatments are in the pipeline towards ground-breaking clinical therapies. We conclude that dentistry has come a long way apropos of regenerative medicine; still, there are vast avenues to endeavour, seeking inspiration from other facets in biomedical research.

## Figures and Tables

**Table 1:** Ion-release-based biomaterials for dentin remineralisation.

Material	Modifications and Use	Ref.
Bioactive glass (BAG)	Modified with zinc, copper, fluoride, and PAA (polyacrylic acid), used as adhesive agents in dentin resin bonding interfaces, and used in dentin hypersensitivity treatment.	[27,28,29]
Calcium silicates	Di/tricalcium silicates and Mineral trioxide aggregate (MTA); bioactivity by alkalisation of hydroxyl groups from the CaOH phase, leading to an increase in pH, decreased activity of MMP, activated mineral precipitation, and antimicrobial activity.	[30]
Calcium orthophosphates	Modifications based on different concentrations of Ca–P, dicalcium phosphate anhydrous (DCPA), dicalcium phosphate dihydrate (DCPD), and tetra calcium phosphate (TTCP) as resin adhesives for dentin remineralisation and improved dentin resin interfaces.	[31]
ZnO particles	Zinc oxide and Zn-loaded polymeric nanomaterials additives in resin, protective action in collagen degradation, and initiation of precipitation of poorly crystallised apatite crystals.	[32,33]

**Table 2:** Biomimetic analogues for dentin remineralisation.

Biomimetic Analogues	Modifications and Use	Ref.
Polyacrylic acid9 (PAA9)	Calcium-binding molecule analogous to dentin matrix protein 1(DMP 1) stabilises and controls dimensions of calcium carbonate and calcium phosphate phases.	[34,35]
Polyvinyl phosphonic acid (PVA)	Functions analogous to collagen-binding matrix phosphoproteins like DMP1 and dentin phosphoproteins.	[35,36]
Sodium trimetaphosphate (STMP)	It is a phosphophoryn analogue, binds to collagen fibrils, creates negatively charged sites to receive nanoprecursors, and initiates nucleation of apatite crystals.	[34,37]
Polyaspartic acid (PAS)	Is an analogue for calcium utilisation, released from hardened calcium silicate cements or calcium phosphate mineralising solutions, and assists in controlling the size of ACP nano precursors and their movement into the collagen fibrils.	[38,39]

**Table 3:** Cell-free therapies for dentin and pulp regeneration.

Biomaterial	Indication/Mechanism/Results	Ref.
Calcium hydroxide (CaOH_2_)	It is a gold standard, with high pH inducing necrosis and mineralisation, good antibacterial properties, and formation of heterogeneous dentin bridge with tunnel defects; it increases recruitment, migration, proliferation, and mineralisation of DPSCs and periodontal ligament stem cells (PDLSCs) through the expression of STRO-1 and CD146 markers; and calcium increases the synthesis of biomolecules such as fibronectin and bone morphogenetic proteins (BMPs) and causes precipitation mineralisation.	[43,44,45,46,47]
Mineral trioxide aggregate (MTA)/Calcium silicates/modifications	It has an antibacterial effect by releasing calcium hydroxide, a superior sealing ability, low solubility, higher strength, and more stability than CaOH; it works well in a moist environment; it forms thicker dentin bridges; it has less inflammatory response, hyperaemia and lower pulp tissue necrosis; modifications include calcium chloride additions, leads to lower setting time, and more biocompatibility; light cured, resin-modified calcium-silicate-based MTA provides immediate polymerization, material preservation, and superior physical properties; and it induces generation of proangiogenic factors like IL-8 and IL-beta (interleukins).	[45,48,49,50,51,52]
Bioactive glasses	It is a mixture of silica, sodium, and phosphorous oxides with the ability to bond to bone by controlled release of ions forming apatite crystals repairing hard tissues; it mimics the natural apatite structure; studies show dentin bridge formation on pulp capping, no necrosis of pulp tissue, and mild inflammatory response; it can form different qualities of reparative dentin with varying porosities and mechanical properties; and it is noncytotoxic and improves cell metabolic activities on in vitro testing.	[53,54]
Biodentine^TM^	Induces differentiation of DPSCs by MAPK (mitogen-activated protein kinase) and calcium calmodulin-dependent protein kinase II (CaMKII) pathways; faster mineralisation of pulp tissue due to the release of transforming growth factor (TGF- Beta 1).	[55,56]
CEM (Calcium enriched mixture)	It has dentinogenic, cementogenic, and osteogenic properties; they increase the expression of fibroblast growth factor 4 (FGF-4) and bone morphogenetic protein 2 (BMP-2), which favours remineralisation and regeneration.	[57]
Glass ionomers and adhesive resins	It has a proliferative effect on pulp tissue comparable to CaOH when used as a lining material for dentin pulp regeneration, no noticeable antibacterial effect, more inflammatory response seen on pulp, upregulation of fibroblasts and endothelial cells, and an inhibitory effect on Hohl cells; in mechanically injured pulp tissue HEMA (hydroxyethyl methacrylate), it induces secretion of proangiogenic factors like vascular endothelial growth factors (VEGF) and decreases expression of FGF-2; and concerns remain regarding the efficiency and quality of tertiary dentin formation after pulp injury.	[58,59,60]
Enamel matrix derivatives (EMD)	It is shown to be more effective than CaOH and MTA in differentiation and proliferation of human tooth germ stem cells; it is highly biocompatible and has known chemotactic effect and angiogenic effects; studies indicate their use for periodontal regeneration; and it is inversely shown to cause more inflammation on pulp tissue with little or less hard tissue formation when compared with CaOH application.	[61,62,63]

**Table 4:** Cell-based therapies for dentin pulp complex regeneration (cell transplantation strategies).

Cells	Indications/Mechanism/Result	Positive Markers	Negative Markers	Ref.
Stem cells from apical papilla (SCAP)	Present next to immature tooth root apex; remains active even in cases of pulp infections or necrosis due to collateral blood supply; has the potential to differentiate into odontoblast like cells; and shows increased telomerase activity, higher resistance to infection, faster multiplication, and migratory efficiency within root canals	CD49d, CD51/61, CD56, CD73, CD90, CD105, CD106, CD146, CD166	CD14, CD18, CD34, CD45, CD117, CD150	[65,66,67,68]
Dental pulp stem cells (DPSC)	Derived usually from human third molar pulp tissue; has high proliferation and colony-forming ability as dense calcified structures; can differentiate into osteoblasts, odontoblasts, adipocytes, and chondrocytes; and can be used as stem cells in neural disorders due to their ability to induce axonal guidance and differentiate into functional neural cells.	CD9, CD10, CD13, CD29, CD44, CD49d, CD59, CD73, CD90, CD105, CD106, CD146, CD166	CD14, CD31, CD34, CD45, CD117, CD133	[69,70,71,72,73,74,75,76,77]
Stem cells from human exfoliated deciduous teeth (SHEDs)	SHED can form bone, dentin, and differentiate into other nondental mesenchymal cell derivatives; it has a higher proliferation rate that DPSC and bone marrow-derived MSCs, faster population doubling, and osteoinductive properties; and transplantation has shown the architecture and cellularity of tissue formed by SHED to resemble dental pulp closely.	CD13, CD44, CD73, CD90, CD105, CD146	CD14, CD19, CD34, CD43, CD45	[78,79,80,81,82]
Dental follicle stem cells (DFSC)	It has ectomesenchyme-derived connective tissue surrounding enamel and dental papilla; contains progenitors for cementoblasts, osteoblasts, and PDL; and exhibits the ability to differentiate into PDL fibroblasts, to secrete collagen, and to consequently interact with fibres in the bone and cementum surface, and DFSC from human third molars in vitro shows rapid growth and expresses stem cell markers, including Nestin and Notch 1.	CD9, CD10, CD13, CD29, CD44, CD49d, CD59, CD73, CD90, CD105, CD106, CD166	CD31, CD34, CD45, CD133	[75,83,84,85,86,87]

**Table 5:** Scaffold-based regeneration of the dentin-pulp complex (cell homing strategies).

Scaffold	Indications/Mechanism/Results	Ref.
Intracanal blood clot	It induces apical bleeding, leading to delivery of SCAP to root canal space; it is an autologous scaffold with growth factors, is clinically efficient, and is economical; and it can be an unstable and unreliable movement of stem cells within the canal space after revascularization.	[88,89,90,91]
Platelet-rich plasma (PRP)	Autologous injectable scaffold: it can be delivered via collagen sponges; platelet elevation results in increased production and secretion of growth factors PDGF, TGF-b, Insulin-like growth factor (IGF), epidermal growth factor (EGF), and epithelial cell growth factor (ECGF), leading to improved angiogenesis and cell proliferation	[88,92]
Alginate	Natural polysaccharides from cell walls and seaweeds: stem cells can be incorporated during scaffold processing; it supports 3-D printing in combination with proteins like DMPs; it includes easy diffusion of nutrients and waste debris due to porous structure; it is highly biocompatible, has low immune reactions, is economic, and is easy to fabricate; and has low mechanical strength of the scaffold when used alone.	[93,94]
Hyaluronic acid (HA)and derivatives	Glycosaminoglycans which mimic ECM components: it interacts with stem cell receptors and drives them towards the area of regeneration; it is shown to have a role in dentin matrix and pulp tissue development; it exhibits good biocompatibility, biodegradability, and bioactivity; HA derivatives induce proangiogenic factors release; it improves stem cell mineralisation and odontogenic differentiation; it has low mechanical strength and needs combination with growth factors to improve regenerative potential; and it may cause hypersensitivity reactions.	[90,94,95,96,97]
Chitosan derivatives	Linear amino polysaccharide mimics ECM structure and composition: it is easy to fabricate, is highly porous, and allows easy migration of cells and growth factors; when fabricated as nanoparticles, it improved properties due to increased surface area, has better mechanical strength, and is resistant to enzymatic degradation; it allows the controlled release of growth factors and improves stem cell or SCAP adhesion, viability, and differentiation; and it is highly biocompatible, has controlled biodegradation, and has low cytotoxicity with antibacterial properties.	[98,99,100]
Gelatin	Consists of proteins from hydrolysis of hard and soft tissue-derived collagen; they are biocompatible and biodegradable, elicits no immune responses, and is cost-efficient; they can be modified with RBDs (receptor binding motifs), which promotes cell attachment and allows chemical modifications to improve the scaffold’s physiochemical properties; it is used as a drug delivery medium or in 2D and 3D cultures; and the use of FGF-2 with gelatin shows the formation of osteo-dentin-like calcified tissue for dentin pulp complex regeneration.	[101,102]
Cellulose	Naturally occurring scaffold obtained from green plants and algae: they are not biodegradable due to the absence of cellulase enzymes in humans; they possess high tensile strength, high crystallinity, fine fibrous structure, and good formability and is biocompatible; they have higher chances of immune response; and they are used mostly in target-specific drug delivery or growth factor release in dental tissue engineering.	[103,104]
Collagen	It is a natural biomaterial, is easily adapted to root canal morphology, and mimics ECM; the most used is type I, suitable for DPSCs proliferation and mineralisation; it is biocompatible, provides bioactivity by facilitating adhesion and attachment of stem cells, and induces signalling pathways that promote differentiation; the highly porous structure allows easy cell seeding for site-specific delivery; and commercially available SynnOss (bovine type 1 collagen) in conjunction with revascularization forms mineralised cementum-like tissues.	[105,106,107,108,109]
Self-assembling peptide hydrogels -Puramatrix	Synthetic, biocompatible, biodegradable, nontoxic, 3D matrix gel available as a liquid phase, which solidifies when in contact with a physiologic salt environment: in vitro studies show pure matrix support DPSC cell proliferation and viability when evaluated over three weeks within tooth slices; puramatrix showed better in vitro results in terms of cell viability and odontogenic differentiation when used with a co-culture of DPSC/HUVEC (human umbilical vein endothelial cells).	[110,111,112,113]
Poly L- Lactic acid (PLLA) nanofibrous microspheres	Injectable scaffold with integrated BMP-2, when combined with polylactic acid (PLA) and polyglycolic acid (PGA), significantly improving the properties and half-life of the PLLA and prolonged BMP-2 release: it is easily adapts to root canal shape and is biodegradable; it can incorporate drugs/growth factors and is conductive for cells, including DPSC and SHED; it has favourable viscosity and porosity; it does not elicit any adverse immune response; it is cheap and reproducible; the regenerated dentin structure may be disorganized and may not replicate the natural tooth architecture; and degradation metabolites might cause unfavourable conditions for surrounding cells but can be excreted to urine without complications.	[114,115,116]
Poly (lactide-co gylcolide)-polyethylene glycol (PLGA-PE) NP	It has better conductivity for dental pulp fibroblasts proliferation; it is clinically biodegradable, has fast setting, has low toxicity, has good biocompatibility, and has low immunogenicity; but, it lacks intrinsic signalling abilities and is more expensive than other synthetic scaffolds.	[106,117]

**Table 6:** Different types of natural bone grafts.

Type of Graft	Action	Advantage	Disadvantage
Autograft	Osteogenic Osteoinductive Osteoconductive	Histocompatible Negligible immunogenicity Ideal physical and mechanical properties	Donor site injury Scarring Longer recovery time Limited size
Ectopic prefabrication	Osteogenic Osteoinductive Osteoconductive	Histocompatible Negligible immunogenicity Ideal physical and mechanical properties No shape or volume limitation	Donor site injury Scarring Longer recovery time
Allografts	Osteogenic Osteoconductive	Histocompatible Ideal physical and mechanical properties.	Immune reaction Transmission of infection
Xenografts	Osteogenic Osteoconductive	Histocompatible Ideal physical and mechanical properties.	Immune reaction Transmission of infection

**Table 7:** Maxillary sinus lift using different bone grafts in oral and maxillofacial surgery.

Ref.	Type of Study	Type of Graft	Method of Evaluation—In Vitro/In Vivo	Sample Size	Conclusions
[193]	Randomized clinical trial (NCT03496688)	MCBA FDBA ABB EB HA-TCP-30/70 BC	Histological and histomorphometric analysis	6 patients	All materials showed good biocompatibility and Osseo conductivity with FDBA as the best material, but only one patient per sample was used, so a larger sample size is required.
[194]	Randomized split-mouth study (NCT03682315)	ACB + ABB ACB + BP	Radiographic analysis, mRNA analysis, histopathological analysis, Immunohistochemistry, TEM	8 patients	Biphasic psychogenic biomaterial (BP) induced a higher radiographical vertical resorption and graft collapse in comparison with the combination with an organic bovine bone (ABB).
[195]	Randomized clinical trial	DPBM vs. DBBM	CT and trephine biopsy histology	11 participants for PPA, 12 ITT	Porcine bone (DPBM) showed comparable results with the widely used bovine bone (DBBM). A larger sample size and more extended studies are still required.
[196]	Randomized clinical trial	MBS BBS	Histological Histomorphometric CBCT	60 patients	BBS remains more stable in terms of volume maintenance and radiological graft homogeneity after a healing period of 6 months.
[197]	Randomized clinical trial	Calcium phosphate crystal double- coated bovine bone and an organic bovine bone	Histological Histomorphometric radiographic	33 patients	Both materials showed comparable histomorphometric and radiographic results.
[198]	Randomized split-mouth study (NCT03077867)	NHA ABB	Histomorphometric	28 patients	After six months of healing, no statistically significant difference was present in histomorphometric outcomes between the NHA and ABB groups.

Mineralised solvent-dehydrated bone allograft (MCBA), freeze-dried mineralised bone allograft (FDBA), anorganic bovine bone (ABB), equine-derived bone (EB), synthetic micro-macroporous biphasic calcium-phosphate block consisting of 70% beta-tricalcium phosphate and 30% hydroxyapatite (HA-TCP 30/70), or bioapatite-collagen (BC); Bio-Oss^®^Spongiosa (Autogenous cortical bone (ACB) + Autogenous Bovine Bone (ABB)) Symbios^®^ Biphasic BGM (ACB + Biphasic psychogenic(BP)); Transmission Electron microscopy (TEM); Deproteinized porcine bone mineral (DPBM), demineralised bovine bone mineral (DBBM), Per-protocol analysis (PPA), Intention to treat analysis (ITT); monophasic bone substitute (100% ß-TCP) (MBS); a biphasic bone substitute (60% HA and 40% ß-TCP) (BBS); Pure sintered nanohydroxyapatite (NHA); and anorganic bovine bone (ABB).
